# A Class II Glutamine Amidotransferase FgDUG3 Is Involved in the Differentiation and Full Virulence of *Fusarium graminearum*

**DOI:** 10.3390/jof11110763

**Published:** 2025-10-23

**Authors:** Chang Su, Peina Cao, Ye Dong, Wenjie Xu, Chenjingzi Hao, Aiguo Gu, Zhengwu Fang, Teng Fu, Dongfang Ma

**Affiliations:** 1 MARA Key Laboratory of Sustainable Crop Production in the Middle Reaches of the Yangtze River (Co-Construction by Ministry and Province)/Hubei Key Laboratory of Waterlogging Disaster and Agricultural Use of Wetland, College of Agriculture, Yangtze University, Jingzhou 434025, China; 2022730075@yangtzeu.edu.cn (C.S.); 2021720806@yangtzeu.edu.cn (Y.D.); 201504436@yangtzeu.edu.cn (W.X.); 19888482232@139.com (C.H.); fangzhengwu88@163.com (Z.F.); 2 State Key Laboratory for Crop Stress Resistance and High-Efficiency Production, College of Plant Protection, Northwest A&F University, Yangling 712100, China; 2024060141@nwafu.edu.cn; 3 Jiangsu Product Quality Testing & Inspection Institute, 5 Guanghua Street, Nanjing 210007, China; nj180618@163.com

**Keywords:** *Fusarium graminearum*, DUG3, transcriptomic analysis

## Abstract

*Fusarium* head blight caused by *Fusarium graminearum* is a serious fungal disease on wheat and maize worldwide, resulting in a significant economic loss. DUG3-mediated glutathione utilization has been revealed to play important roles in fungal differentiation, metabolism, stress adaptation, and plant infection. However, functional roles of the DUG3 homolog in *F. graminearum* remain uncharacterized. In the present study, *FgDUG3* was knocked-out via homologous recombination to investigate functions of this gene. The deletion mutant (Δ*FgDUG3*) was normal in mycelial growth, but showed impairments in conidiation, conidial germination, and plant infection, compared to the wild-type strain. The defects of Δ*FgDUG3* were recovered in the complemented strain (Δ*FgDUG3-C).* Transcriptomic analysis revealed that deletion of *FgDUG3* caused significantly differential expression of genes, mainly related to metabolism, catabolism, cellular structure organization, and signal transduction. Taken together, these results suggest that *FgDUG3* plays important roles in the differentiation and pathogenicity of *F. graminearum*.

## 1. Introduction

*Fusarium graminearum* is a globally distributed filamentous fungus and a major causal agent of Fusarium head blight on cereal crops, particularly maize and wheat barley [1,2]. This pathogen poses a significant threat to global food security by reducing crop yields and grain quality [3]. Beyond its agricultural impact, *F. graminearum* is notorious for producing harmful mycotoxins, especially deoxynivalenol (DON), which contaminate food and feed, posing serious health risks to humans and animals [4,5]. The fungus exhibits high genetic diversity and adaptability, with a complex life cycle that involves survival in crop residues, soil, and seeds, facilitating both local persistence and long-distance dispersal.

Glutathione (GSH), a ubiquitous tripeptide composed of γ-glutamyl, cysteinyl, and glycine residues, functions as a stress metabolite across a wide range of prokaryotes and eukaryotes [6,7]. GSH not only acts as the primary redox stabilizer to resist heavy metals, xenobiotics, and reactive oxygen species (ROS), but also serves as a nitrogen and sulfur reservoir [8,9,10]. In plant-pathogenic fungi, GSH homeostasis is tightly linked to virulence, enabling the pathogens to mitigate oxidative bursts from host immune responses [11]. Moreover, GSH plays an important role in supporting fungal growth and survival under nutrition-restricted conditions [12]. It is well established that GSH is biosynthesized by GSH synthetases, and biodegraded by γ-glutamyl transpeptidase [13].

However, some fungal organisms have evolved alternative pathways to utilize GSH, which is not dependent on γ-glutamyl transpeptidase. For example, the disruption mutant of *Saccharomyces cerevisiae* lacking γ-glutamyl transpeptidase grew well with glutathione as the sole sulfur source, proving the existence of an alternative pathway [14]. Further research confirmed that a protein complex, containing DUG1, DUG2, and DUG3, mediates the degradation of GSH in *S. cerevisae* [15]. *DUG3* encodes a glutamine amidotransferase-like protein, acting as a central component of the glutathione degradation complex. Unlike the extracellular γ-glutamyl transpeptidase pathway, the DUG pathway allows fungi to recycle GSH directly in the cytoplasm, particularly under nutrient stress conditions. The loss of function of DUG3 leads to glutathione utilization defects, making cells unable to use GSH as a sole sulfur or nitrogen source.

Although the DUG3 orthologs are present in a wide range of fungi, their functional roles have been characterized in a few species. For example, the DUG3 homolog has been revealed to play important roles in stress adaptation, metabolism, conidiogenesis, and sexual differentiation in *Aspergillus nidulans* [7]. In the rice blast fungus *Magnaporthe oryzae*, the disruption of the *DUG3* homolog caused defects in conidiation, appressorium development, and plant infection [16]. Although *F. graminearum* has been studied for the roles of glutathione-related genes in antioxidant stress response and pathogenicity [11,17], the functional roles of DUG3 remain unknown in this fungus.

In this study, we aimed to investigate the functions of *FgDUG3* in *F. graminearum* using deletion mutants and transcriptomic analysis. The results showed that the deletion of *FgDUG3* does not affect mycelial growth and sexual differentiation, but caused defects in conidiation, conidial germination, and plant infection. Transcriptomic analysis revealed that deletion of *FgDUG3* resulted in significantly differential expression of genes associated with metabolism, catabolism, cellular structure organization, and signal transduction.

## 2. Materials and Methods

### 2.1. Fungal Strains and Culture Conditions

The *F. graminearum* strain PH-1 was used as the wild-type strain in this study. The strain PH-1 was kindly provided by Prof. Huaigu Chen and his Wheat Disease Control research team at the Institute of Plant Protection, Jiangsu Academy of Agricultural Sciences, China.

In this study, the wild-type strain, knockout mutants, and complemented strains were regularly cultured on potato dextrose agar medium (PDA) and complete agar medium (CM) for the evaluation of mycelial growth as previously described [18]. Mycelia were inoculated to carboxymethyl cellulose (CMC) liquid medium and cultured in a shaker at 150 rpm and 25 °C for producing conidia [19]. The CMC liquid medium was used for conidial germination, following a previous method [20].

### 2.2. Bioinformatic Analysis

The amino acid sequences used in this study were downloaded from GenBank (https://www.ncbi.nlm.nih.gov/genbank/, accessed on 1 March 2024). The domain of selected proteins was predicted using InterProScan (https://www.ebi.ac.uk/interpro/search/sequence/, accessed on 1 March 2024). Phylogenetic analysis of FgDUG3 and its homologs was performed using MEGA12 (https://www.megasoftware.net/, accessed on 1 March 2024).

### 2.3. Target Gene Deletion and Complementation

The split-marker method was used to generate a knockout mutant of the FgDUG3 gene [21]. Briefly, the upstream and downstream fragments of FgDUG3 gene were amplified from genomic DNA of a PH-1 strain with paired primers 1F/1R and 2F/2R, respectively (Table A1). The upstream fragment hph-F and downstream fragment hph-R of hph cassette were amplified with paired primers HYG/F/HY/R and YG/F/HYG/R, respectively, as described previously [19]. The upstream fragments of FgDUG3 gene and upstream fragments of hph cassette were fused and amplified with paired primers 1F/HY/R (Table A1). Similarly, both downstream fragments were fused and amplified with paired primers YG/F/2R (Table A1). The two purified fusion segments were introduced into protoplasts of PH-1 strain using a PEG-mediated transformation [19]. The transformants were grown on medium amended with hygromycin B, and then screened with paired primers 5F/6R and H856F/H855R (Table A1).

For complementation, the construct containing the *FgDUG3* open reading frame and the upstream and downstream fragments were transformed into protoplasts of Δ*FgDUG3*. Transformants of deletion mutants and complemented strains were initially selected on TB3 agar media containing 200 µg/mL G418, respectively, and next screened by PCR with paired primers 5F/6R, 3F/3R, and 4F/4R (Table A1).

### 2.4. Characterization of Phenotypes

For the evaluation of mycelial growth, fungal strains were grown on PDA at 25 °C for two days [22]. Mycelial discs from PDA were inoculated to new PDA and cultured at 25 °C for five days to evaluate colony diameters. For the examination of conidiation and conidium morphology, mycelia obtained from PDA were inoculated to CMC liquid medium and rotated at 150 rpm and 25 °C for five days. The conidia were harvested by filtering through a single layer of sterile filter paper. Then, concentrations of conidial suspensions were evaluated using a hemocytometer. Conidial morphology was examined using a fluorescence microscope (Nikon DS-Ri2, Tokyo, Japan). These experiments were performed on three independent experiments with at least three replicates per experiment. The significant difference was estimated using Tukey’s HSD test (*p* < 0.05).

### 2.5. Sexual Reproduction

The designated strains were cultured on carrot agar at 25 °C for 7 days. Aerial mycelia were scraped off using a sterile medical spoon and cultured under black light at 25 °C. Once the mycelia began to grow, 1% Tween was applied to adhere the mycelia to the medium. After two weeks, photographs were taken using a Leica M205 FA stereoscope (Wetzlar, Germany), with ten replicates for each strain [23]. For ascospore observation, mycelial plugs (10 mm in diameter) containing ascus structures were bisected and placed vertically on the surface of a fungal culture block in a disposable Petri dish, followed by incubation at 25 °C for 24–48 h. The discharged asci were collected from the medium, transferred to a glass slide, and gently pressed under a coverslip. Ascospore morphology was examined using a Nikon ECLIPSE Ni–U microscope equipped with a Nikon DS–Ri2 digital camera (Tokyo, Japan) [24].

### 2.6. Pathogenicity Assay

To evaluate pathogenicity in flowering wheat heads, conidia were harvested from *Fg* strains after four days of cultivation in CMC liquid medium, resuspended in 0.01% (*v*/*v*) Tween 20, and adjusted to a final concentration of 10^5^ conidia/mL. For pathogenicity in wheat spikelets, the prepared conidial suspensions (10 µL) were injected into grains in the middle wheat spikelet. For the control, wheat grains were inoculated with sterile distilled water (SDW) through the same method described above. Next, the bag was sprayed with SDW to maintain humidity for two days. At 14 days post-inoculation, the number of symptomatic spikelets exhibiting blight symptoms on each wheat head was assessed [20].

For pathogenicity on corn silks, sterile filter papers were placed in a glass dish, and moistened with SDW. Using a sterile blade, both ends of four corn silks were cleanly cut and laid flat on the moistened filter papers. Five replicates were applied for each strain. The inoculated corn husks were placed in an incubator at 25 °C [18]. After four days, the lesion sizes were measured and photographs were taken for record.

### 2.7. Transcriptomic Analysis

**Figure A1:**
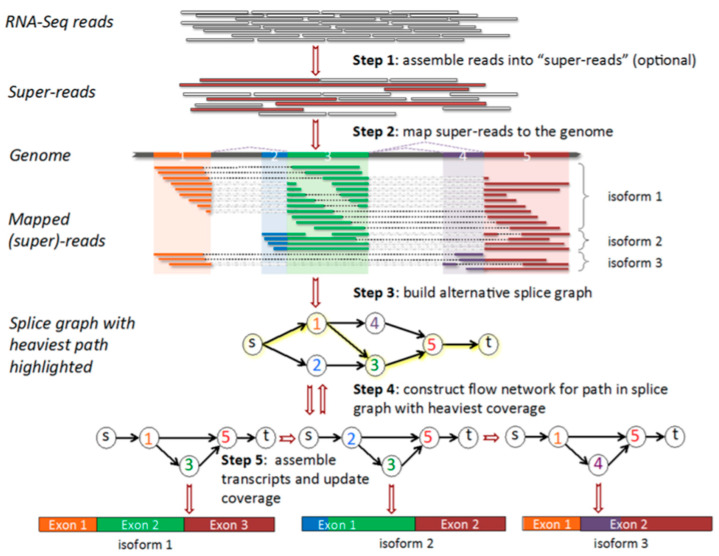
An analysis pipeline for assembling, mapping, and annotation of RNA-seq reads. The numbers 1, 2, 3, 4, and 5 within the circles represent exons.

For transcriptome profiling, wheat heads were injected with conidial suspensions of the strains PH-1 and Δ*FgDUG3*, respectively (Genome Sequence Archive, CRA030539). Inoculated spikelets were harvested at 7 dpi, and immediately frozen in liquid nitrogen for total RNA extraction. Three biological replicates of extracted RNA samples were sequenced by Illumina Hiseq 2500 [25]. The RNA-seq reads were filtered, mapped, and assembled with a reference genome of *F. graminearum* PH-1, according to a pipeline (Figure A1). The distribution and mapping of total reads were shown in Table A2 and Table A3. The differentially expressed genes (DEGs) between PH-1 and Δ*FgDUG3* were identified using DEGSeq2 with a threshold of |log2 fold change| > 1 and *p* < 0.05.

## 3. Results

### 3.1. Phylogenetic Analysis of DUG3 Proteins

**Figure 1:**
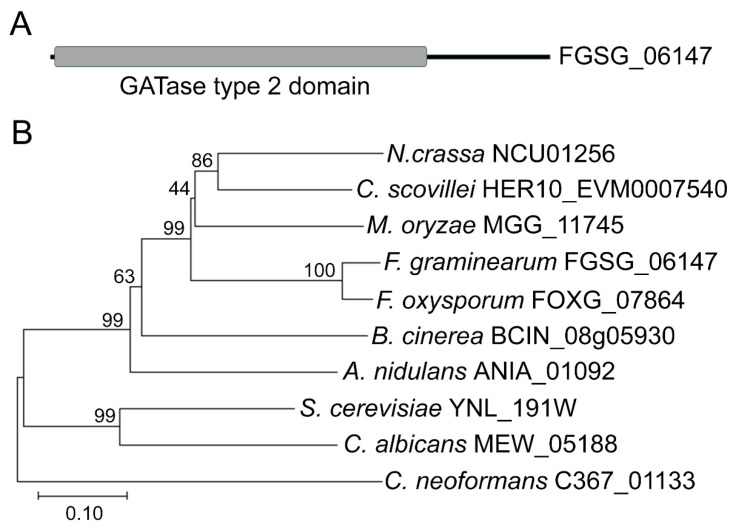
Domain representation and phylogenetic relationship. (**A**) Domain representation. Black line and gray box represent amino acid sequence and GATase type 2 domain (IPR017932), respectively. (**B**) Phylogenetic relationship. The phylogenetic tree was constructed based on FgDUG3 and its homologs using the neighbor-joining method with 1000 bootstraps. The full Latin names are as follows: *N. crassa* (*Neurospora crassa*), *C. scovillei* (*Colletotrichum scovillei*), *M. oryzae* (*Magnaporthe oryzae*), *F. graminearum* (*Fusarium graminearum*), F. oxysporum (Fusarium oxysporum), B. cinerea (Botrytis cinerea), *A. nidulans* (*Aspergillus nidulans*), *S. cerevisiae* (*Saccharomyces cerevisiae*), *C. albicans* (*Canidia Albicans*), *C. neoformans* (*Cryptococcus neoformans*).

Using the amino acid sequence of *S. cerevisiae* DUG3 (YNL_191W) as a query search in the genome of *F. graminearum* PH-1, we identified a sequence FGSG_06147 (named FgDUG3). The *FgDUG3* gene is predicted to encode a protein (476 amino acids) comprising a glutamine amidotransferase type 2 domain (Figure 1A). To analyze phylogenetic relationships, a neighbor-joining tree was constructed based on FgDUG3 and its homologs from other selected fungi. FgDUG3 was found to be closely related to its homologs from *Fusarium oxysporum*, and distantly related to homologs from *S. cerevisiae*, *Candida albicans*, and *Cryptococcus neoformans* (Figure 1B). This result suggests that FgDUG3 is conserved among fungi.

### 3.2. Generation of FgDUG3 Deletion Mutants

**Figure 2:**
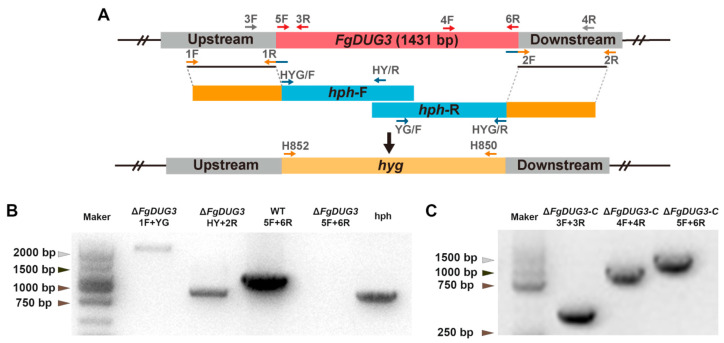
Detection of *FgDUG3* knockout and complementation transformants. (**A**) Schematic of gene knockout via homologous recombination. (**B**) Four pairs of primers PCR to detect transformant electrophoresis results. (**C**) Positive transformants of the *FgDUG3* complementation strain were screened by PCR.

To characterize the functions of FgDUG3, a split-marker method was used to generate the Δ*FgDUG3* (Figure 2A). To restore *FgDUG3* gene function, complementation strains Δ*FgDUG3-C* were constructed by introducing the native promoter-driven coding sequence of *FgDUG3* under the control of its native promoter-driven coding sequence of *FgDUG3*. PCR was performed to verify the genotypes of Δ*FgDUG3* and Δ*FgDUG3-C* (Figure 2B,C). Two independent Δ*FgDUG3* and Δ*FgDUG3-C* strains were generated for subsequent functional analyses. Since the results were highly consistent between biological replicates, data from one representative strain of each genotype are shown herein.

### 3.3. FgDUG3 Is Dispensable for Mycelial Growth

**Figure 3:**
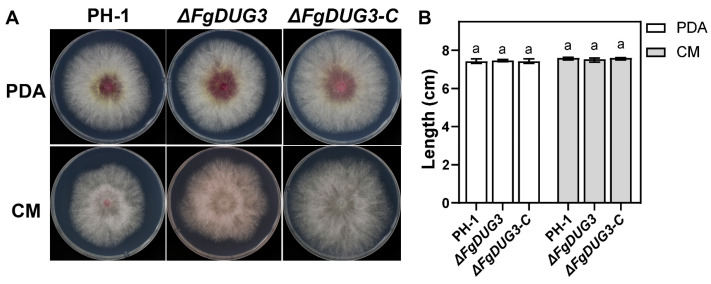
Examination of colony growth. Mycelial growth and colony morphology of the indicated strains were evaluated on PDA and CM. (**A**) Observation of colony diameter. (**B**) Length of colony diameter. The same letter in group indicate significant difference according to Tukey’s HSD test (*p* < 0.05).

To investigate the role of *FgDUG3* in fungal growth, mycelial discs were inoculated onto PDA and CM. After 5 days, colony diameter and morphology were evaluated. Δ*FgDUG3* was found to be normal in mycelial growth rate and colony morphology, compared to the PH-1 and Δ*FgDUG3-C* (Figure 3A,B). This result suggests that *FgDUG3* is dispensable for mycelial growth of *F. graminearum*.

### 3.4. FgDUG3 Is Involved in Asexual Reproduction

**Figure 4:**
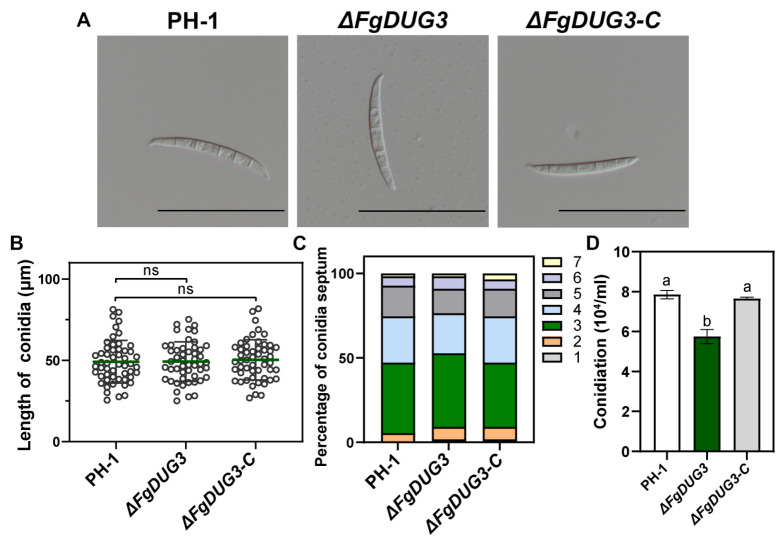
Evaluation of conidiation. (**A**) Conidium morphology. Scale bar = 50 μm. (**B**–**D**) Statistics of conidial size, septation number, and conidial production. Different letters in groups indicate significant difference according to Tukey’s HSD test (*p* < 0.05). ns, no significant difference.

As asexual reproduction plays an important role in the dissemination of *F. graminearum*, we evaluated conidiation and conidium morphology in CMC broth. The Δ*FgDUG3* mutant and its complemented strain (Δ*FgDUG3-C*) showed conidial morphology comparable to the wild-type PH-1, with no significant differences in the mean conidial length (∼50 μm) or in the distribution of septation numbers, where conidia with three septa were predominant (∼40%) (Figure 4A–C). Quantitative evaluation of conidiation showed that PH-1 and Δ*FgDUG3-C* produced (8.2 ± 0.7) × 10^4^ conidia/mL and (8.0 ± 0.6) × 10^4^ conidia/mL, respectively. However, Δ*FgDUG3* produced (6.0 ± 0.5) × 10^4^ conidia/mL, which is significantly less than that of PH-1 and Δ*FgDUG3-C* (Figure 4D).

**Figure 5:**
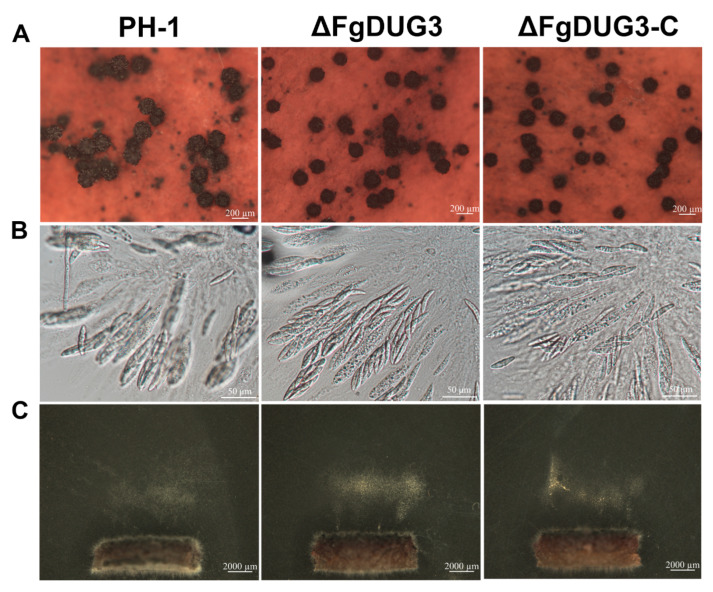
Sexual reproduction. After being cultivated on CA plates for 3–5 days on carrot agar (CA) medium, the mycelia of each strain were gently scraped with a sterilized spoon, and added with 1 mL 0.2% Tween 20 to each dish. The plates were incubated at 18–24 °C under a 12 h light/12 h dark cycle for 7–14 days. (**A**) Photographs of perithecia production. (**B**) Microscopic observation of ascospores. (**C**) Ascospore discharge.

To investigate whether *FgDUG3* is related to sexual reproduction, we inoculated strains on carrot agar medium. Δ*FgDUG3* was found to be normal in the production of perithecia and ascospores, compared to PH-1 or Δ*FgDUG3-C* (Figure 5A–C), revealing that *FgDUG3* is not involved in sexual reproduction. These results suggest that *FgDUG3* is involved in conidiation but not conidium morphology of *F. graminearum*.

### 3.5. FgDUG3 Is Associated with Germ Tube Development

**Figure 6:**
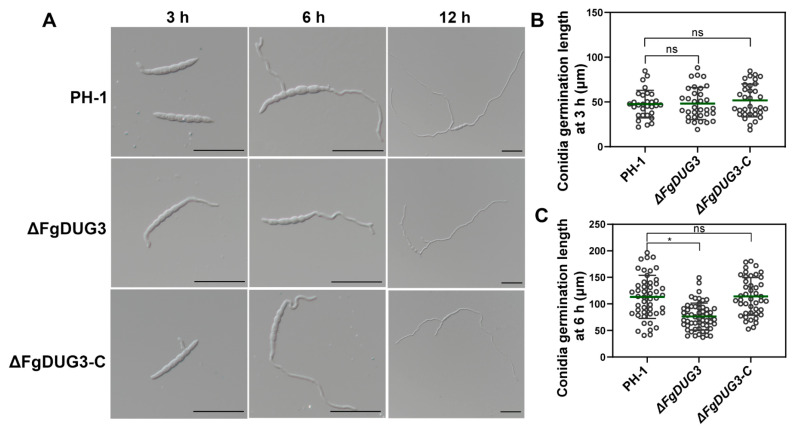
Conidial germination and germ tube morphology. (**A**) Microscopic observation of morphology of germ tube at 3, 6, and 12 h. Scale bar = 50 μm. (**B**) Quantitative measurement of germ tube length at 3 h and (**C**) 6 h. 30 to 60 conidia were examined in each replicate. Statistically significant analyses were based on Student’s *t*-test: ns, no significant difference; *, *p* < 0.05.

The conidial germination rate was found to be normal in Δ*FgDUG3*, compared to PH-1 or Δ*FgDUG3-C* (Figure 6A), suggesting that *FgDUG3* is dispensable for the conidial germination rate of *F. graminearum*. Notably, the germ tube length of Δ*FgDUG3* was significantly shorter than that of PH-1 or Δ*FgDUG3-C* at 6 h (Figure 6B). These results suggest that *FgDUG3* is associated with the germ tube development of *F. graminearum*.

### 3.6. FgDUG3 Is Required for Full Virulence

**Figure 7:**
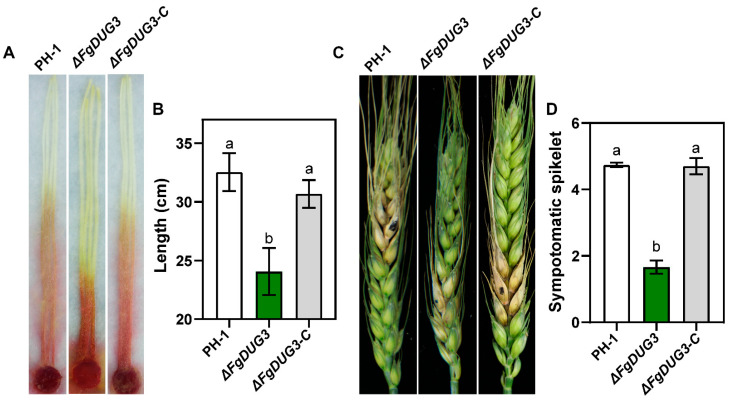
Pathogenicity assays. (**A**,**B**) Lesions developed on corn silks. Mycelial plugs were inoculated to corn silks and incubated for 4 days. Photographs of lesions on corn silks (**A**). Statistical analysis of lesion length on corn silk (**B**). (**C**,**D**) The flowering wheat heads were injected with conidial suspensions (10^5^ conidia/mL) via syringe needles and incubated for 14 days. Photographs of lesions on flowering wheat heads (**C**). Statistical analysis of lesion length on flowering wheat heads (**D**). Three independent biological experiments were conducted. In each experiment, measurements were obtained from 5 to 10 individual corn silks or wheat spikes per biological replicate (*n* = 5–10). Data are presented as the mean ± SEM of three biological replicates and were analyzed by one-way ANOVA. Significant differences (*p* < 0.05) according to Tukey’s test are indicated by different lowercase letters.

To test whether *FgDUG3* is related to the virulence of *F. graminearum*, mycelial plugs were inoculated to corn silks. After 4 d, the lesion length caused by Δ*FgDUG3* was significantly shorter than that of PH-1 or Δ*FgDUG3-C* (Figure 7A,B). We next investigated the role of *FgDUG3* in infection on flowering wheat head by inoculating conidial suspensions. After 14 d, Δ*FgDUG3* infected significantly fewer spikelets than WT or Δ*FgDUG*3-C (Figure 7C,D). These results suggest *FgDUG3* is required for full the virulence of *F. graminearum* on corn and wheat.

### 3.7. Differentially Expressed Genes Regulated by FgDUG3

**Figure 8:**
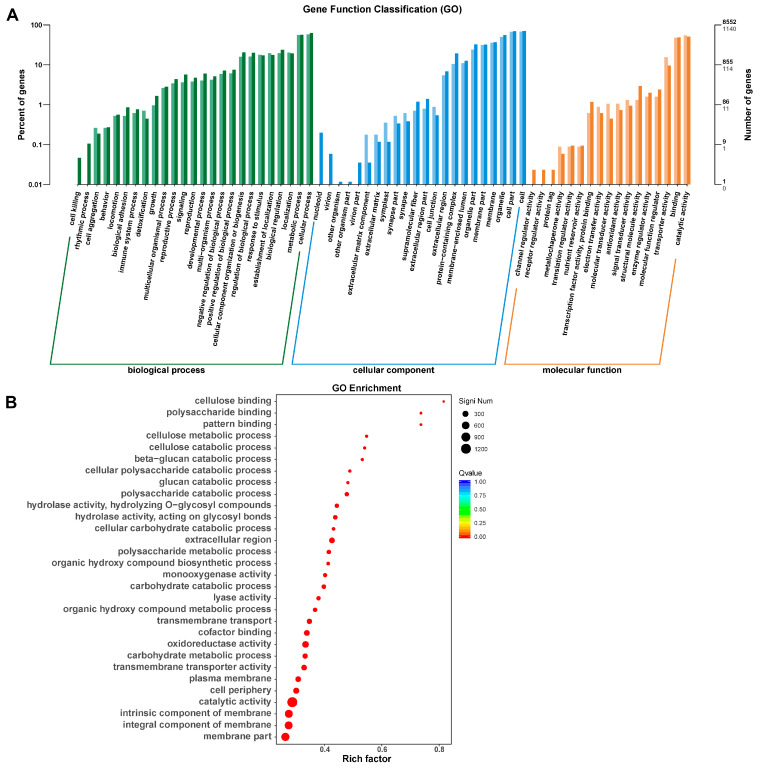
Transcriptomic analysis. The flowering wheat heads were ejected with conidial suspensions (10^5^ conidia/mL) via syringe needles. After 7 days, the gene expression was determined via RNA-seq. (**A**) Classification of differentially expressed genes (DEGs) based on gene ontology (GO). (**B**) GO enrichment of DEGs.

**Figure A2:**
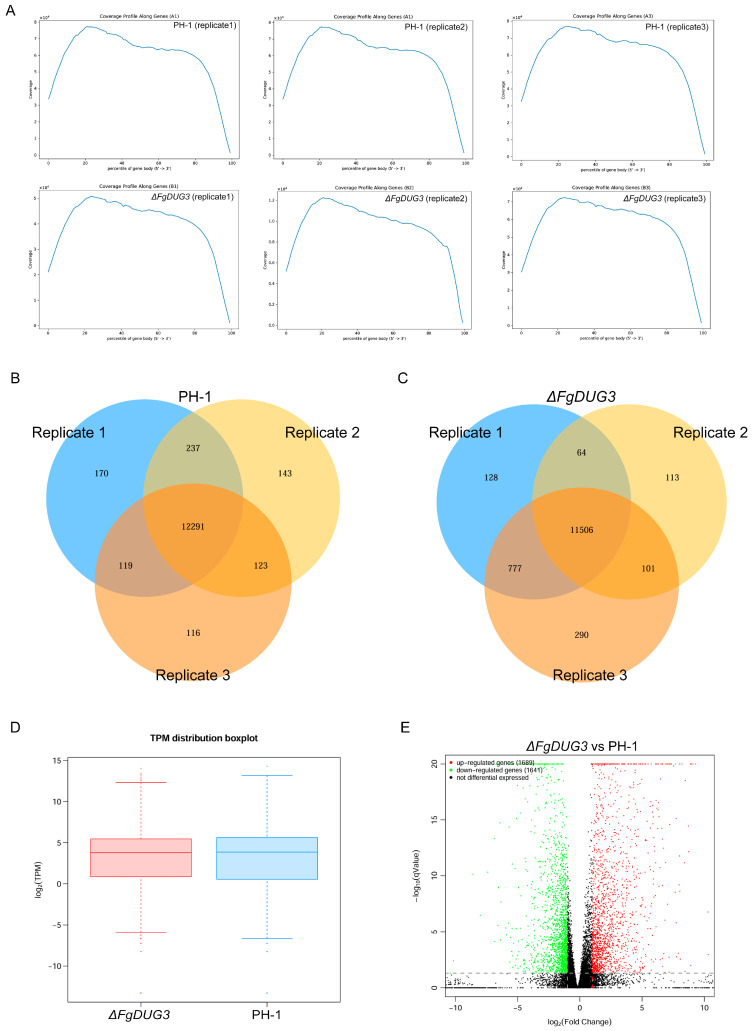
Global analysis of RNA-seq data. (**A**) Coverage profile along genes of six samples. (**B**,**C**) Venn diagram of detected genes among three replicates of PH-1 (**B**) and Δ*FgDUG3* (**C**). (**D**) TPM distribution of detected genes in PH-1 and Δ*FgDUG3*. (**E**) Volcano diagram of differentially expressed genes (DEGs) in Δ*FgDUG3* referenced to PH-1.

To further investigate the genes regulated by FgDUG3 during plant infection, the RNA-seq analysis was performed to profile the gene expression of Δ*FgDUG3* and the PH-1 strain during wheat head infection at 7 dpi. A total of 3330 genes were identified as significantly differentially expressed (*p* < 0.05), with 1689 upregulated genes (log_2_ fold change > 1) and 1641 downregulated genes (log_2_ fold change < 1) (Figure 8 and Figure A2, Table A2). The Gene Ontology (GO) analysis showed that DEGs were classified into biological processes, cellular components, and molecular functions. The GO enrichment analysis revealed that DEGs were enriched in categories related to polysaccharide metabolism, catabolic processes, enzymatic activity, and structural components of the cell (Figure 8A,B).

**Figure 9:**
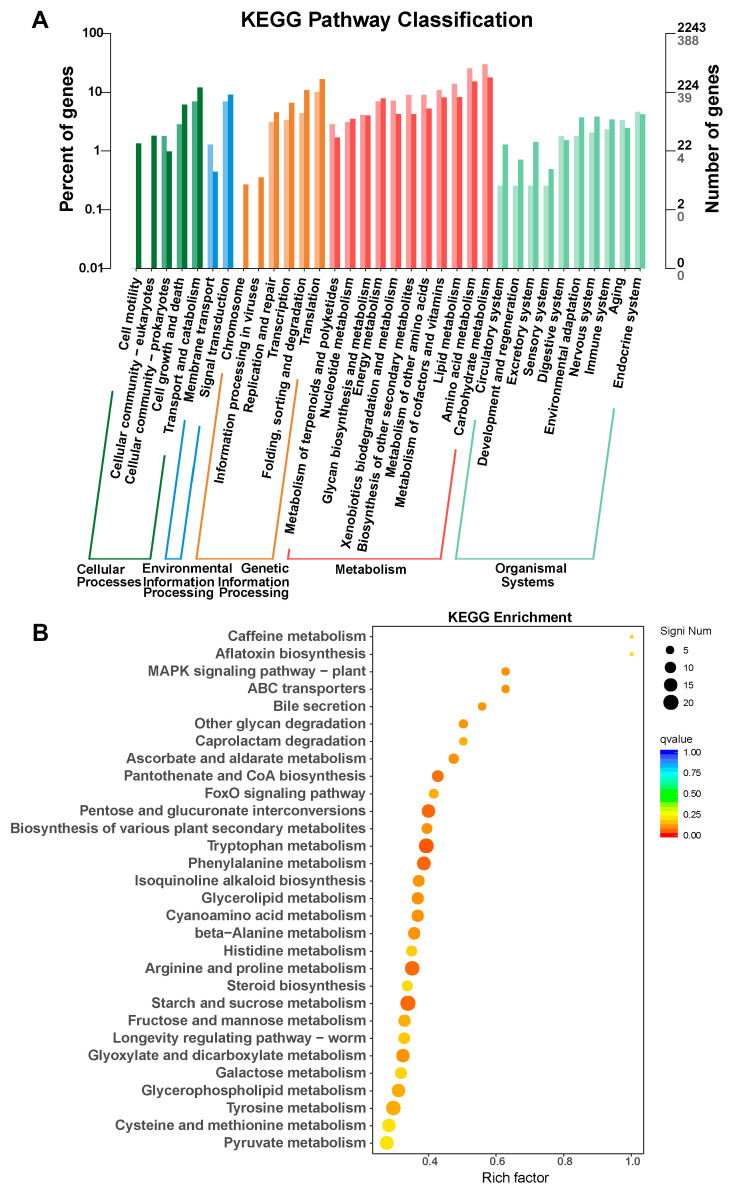
Functional enrichment analysis. (**A**) Classification of DEGs based on Kyoto Encyclopedia of Genes and Genomes (KEGG). (**B**) KEGG enrichment of DEGs.

The Kyoto Encyclopedia of Genes and Genomes (KEGG) enrichment analyses revealed that the DEGs were enriched in signal transduction and multiple metabolic pathways, including caffeine metabolism, starch and sucrose metabolism, and amino acid metabolic pathways (Figure 9A,B). Collectively, these results indicate that the deletion of *FgDUG3* caused the major alterations in metabolism, catabolism, cellular structure organization, and signal transduction.

## 4. Discussion

The present study aims to investigate the functional roles of FgDUG3 in the wheat head blight pathogen *F. graminearum*. Our results demonstrated that *FgDUG3* plays important roles in asexual reproduction, germ tube development, and virulence, while being dispensable for vegetative growth and sexual reproduction. Furthermore, transcriptomic analysis revealed significantly differential expression of genes involved in metabolism, signaling, and structural components following *FgDUG3* deletion. The findings suggest that *FgDUG3* is important for the fungal differentiation and pathogenicity of *F. graminearum*, which would provide novel insight into the molecular mechanisms governing *Fusarium* head blight development.

*FgDUG3* was predicted to encode a protein containing a glutamine amidotransferase type 2 domain (Figure 1A), a conserved enzymatic motif involved in amino group transfer and nitrogen metabolism [26]. In terms of phylogenetic relationships, FgDUG3 is closely related to its homologs from *Fusarium oxysporum*, *M. oryzae*, and *Botrytis cinerea*, and more distantly related to those from *S. cerevisiae*, *Candida albicans*, and *Cryptococcus neoformans* (Figure 1B). This is consistent with the divergence between ascomycete fungal plant pathogens and yeast-like fungi. The conservation of domain architecture indicates that FgDUG3 may retain core enzymatic activities, yet functional divergence is also plausible. In yeast, the DUG pathway contributes to glutathione degradation and nitrogen utilization [13,14]. However, our findings indicate that FgDUG3 additionally regulates developmental processes, such as conidiation and germ tube elongation, as well as pathogenicity in *F. graminearum*. Consistently, functions of DUG3 were reported to be involved in conidiation, appressorium development, and plant infection in *M. oryzae* [16]. Thus, FgDUG3 might integrate metabolic activity with developmental regulation in a plant pathogenic context, reflecting the adaptation of conserved proteins to the specific lifestyle of fungal organism.

The deletion of *FgDUG3* did not affect mycelial growth and morphology on PDA and CM (Figure 3), indicating that vegetative hyphal growth under nutrient-rich conditions is maintained without *FgDUG3*, possibly due to redundancy in glutamine amidotransferase. However, Δ*FgDUG3* significantly reduced conidiation, suggesting that FgDUG3 is involved in regulating asexual reproduction in *F. graminearum*. Importantly, conidial morphology of Δ*FgDUG3* remained unaltered, suggesting that *FgDUG3* specifically influences conidiation rather than morphogenesis. This finding supports that fungal reproduction is tightly linked to nutrient metabolism [27,28,29]. As glutamine amidotransferases participate in amino acid metabolism and nitrogen assimilation [26,30], *FgDUG3* may act as a metabolic regulator for the conidiation of *F. graminearum*. Interestingly, Δ*FgDUG3* was normal in the production of perithecia and ascospores (Figure 5), indicating that sexual reproduction relies on distinct regulatory circuits that bypass FgDUG3. The molecular network regulating asexual and sexual reproduction has been previously documented in *F. graminearum* [31,32,33]. Our findings reinforce the idea that asexual and sexual cycles are governed by partially overlapping but distinct networks, with *FgDUG3* being specific to asexual reproduction.

Δ*FgDUG3* was normal in conidial germination rate, but defective in germ tube elongation (Figure 6), suggesting that FgDUG3 is dispensable for initial polarity establishment but required for sustained polarized growth. Germ tube elongation is a complex process involving cytoskeletal organization, vesicle trafficking, and cell wall remodeling [34,35]. Metabolism is also essential for germ tube elongation, as the synthesis of macro-molecules contributes to tip growth. For example, nitrogen starvation induces morphogenetic transitions critical for infection structure formation in *M. oryzae* [36]. Therefore, the defect in germ tube elongation in Δ*FgDUG3* might arise from metabolic insufficiency or the misregulation of developmental signaling.

Δ*FgDUG3* significantly attenuated virulence on both corn silks and wheat heads, indicating the important function of *FgDUG3* in pathogenicity (Figure 7). During host colonization, fungal plant pathogens encounter nutrient limitations, oxidative stress, and plant defense responses [37,38]. Glutamine amidotransferase may be involved in synthesizing or recycling key metabolites to support infection. The defect of Δ*FgDUG3* in pathogenicity suggests that the metabolic change conferred by *FgDUG3* is essential for host colonization. This was supported by the transcriptomic profiling, as widespread alterations of gene expressions involved in polysaccharide metabolism, amino acid metabolism, and signal transduction (Figure 8). The enrichment of carbohydrate metabolism-related genes is particularly relevant. Successful colonization of host tissues requires the efficient degradation and utilization of plant polysaccharides. Downregulation of such genes in Δ*FgDUG3* mutants may compromise nutrient acquisition, leading to impaired colonization in plants. Similarly, amino acid metabolism is critical for nitrogen assimilation and secondary metabolite biosynthesis. Given that the biosynthesis of trichothecene mycotoxins in *F. graminearum* is nitro-gen-regulated [39], the disruption of nitrogen metabolism through the loss of function of FgDUG3 could indirectly affect secondary metabolism and virulence. Furthermore, enrichment of DEGs in signal transduction pathways suggests that FgDUG3 influences cellular communication and environmental sensing. Metabolic enzymes are increasingly recognized to play a role in signaling [40,41]. Thus, FgDUG3 may serve as both a metabolic enzyme and a regulator of signaling cascades, orchestrating the transcriptional programs essential for adaptation to the host environment.

## 5. Conclusions

In conclusion, *FgDUG3* is involved in regulating asexual reproduction and contributes to the pathogenicity of *F. graminearum*, likely through integrating nitrogen metabolism with developmental and signaling pathways. Its absence impairs germ tube elongation and host colonization, underscoring its adaptive role in fungal pathogenicity while highlighting the metabolic basis of infection-related morphogenesis.

## Data Availability

The transcriptomic data presented in the study are openly available in CNCB at CRA030539.

## Abbreviations

The following abbreviations are used in this manuscript:

DON	Deoxynivalenol
GSH	Glutathione
ROS	Reactive oxygen species
PDA	Potato dextrose agar
DEGs	Differentially expressed genes
CM	Complete agar
CMC	Carboxymethyl cellulose

## Appendix A

**Table A1:** Primers used in the present study.

Primer	Sequence (5′-3′)
FgDUG3-1F	AGTGACGCCAGCAGGATA
Hyg-FgDUG3-1R	TTGACCTCCACTAGCTCCAGCCAAGCCTGATTGCGTGTTACGGAT
FgDUG3-2F	TATGGATCGAGGCAAGGT
Hyg-FgDUG3-1R	GAATAGAGTAGATGCCGACCGCGGGTT
FgDUG3-2R	CCTGTGATGGAAAAGAGG
FgDUG3-5F	ATGTGCCGTTTTCTGGTG
FgDUG3-6R	TCACCCCGCATGGGAGGC
HYG/F	GGCTTGGCTGGAGCTAGTGGAGGTCAA
HYG/R	AACCCGCGGTCGGCATCTACTCTATTC
HY/R	GTATTGACCGATTCCTTGCGGTCCGAA
YG/F	GATGTAGGAGGGCGTGGATATGTCCT
H852	TTCCTCCCTTTATTTCAGATTCAA
H850	ATGTTGGCGACCTCGTATTGG

**Table A2:** Mapping of total reads.

	A1	A2	A3	B1	B2	B3
Total reads	62,670,288(100.00%)	65,215,212(100.00%)	64,694,700(100.00%)	75,654,640(100.00%)	32,819,352(100.00%)	62,749,358(100.00%)
Total mapped	59,858,562(95.51%)	61,760,765(94.70%)	63,100,844(97.54%)	40,018,705(52.90%)	10,521,595(32.06%)	57,468,385(91.58%)
Mutiple mapped	265,266(0.42%)	233,445(0.36%)	270,593(0.42%)	126,563(0.17%)	835,181(2.54%)	206,012(0.33%)
Uniquely mapped	59,593,296(95.09%)	61,527,320(94.35%)	62,830,251(97.12%)	39,892,142(52.73%)	9,686,414(29.51%)	57,262,373(91.26%)
Read-1 mapped	29,879,586(47.68%)	30,870,888(47.34%)	31,526,988(48.73%)	20,012,291(26.45%)	4,856,115(14.80%)	28,732,486(45.79%)
Read-2 mapped	29,713,710(47.41%)	30,656,432(47.01%)	31,303,263(48.39%)	19,879,851(26.28%)	4,830,299(14.72%)	28,529,887(45.47%)
Reads map to ‘+’	29,786,332(47.53%)	30,753,179(47.16%)	31,403,465(48.54%)	19,941,079(26.36%)	4,840,809(14.75%)	28,626,222(45.62%)
Reads map to ‘-’	29,806,964(47.56%)	30,774,141(47.19%)	31,426,786(48.58%)	19,951,063(26.37%)	4,845,605(14.76%)	28,636,151(45.64%)
Non-splice reads	45,069,390(71.92%)	46,582,022(71.43%)	48,344,264(74.73%)	30,475,976(40.28%)	7,369,629(22.46%)	43,701,686(69.64%)
Splice reads	14,523,906(23.18%)	14,945,298(22.92%)	14,485,987(22.39%)	9,416,166(12.45%)	2,316,785(7.06%)	13,560,687(21.61%)
Reads mapped in proper pairs	58,975,354(94.10%)	60,862,436(93.33%)	62,138,088(96.05%)	39,439,416(52.13%)	9,576,482(29.18%)	56,594,788(90.19%)

**Table A3:** Distribution of total reads.

	A1	A2	A3	B1	B2	B3
NC_026474.1	20,227,948	20,774,447	21,217,351	12,787,728	3,032,299	18,272,007
NC_026475.1	13,050,356	13,511,959	13,890,967	9,574,912	2,153,423	13,694,690
NC_026476.1	13,039,232	13,411,780	13,738,661	8,712,263	2,042,997	12,571,361
NC_026477.1	13,265,090	13,819,345	13,970,199	8,810,227	2,123,575	12,715,866
NW_001837897.1	1630	1603	1674	773	186	1081
NW_001837898.1	0	0	1	0	0	0
NW_001837899.1	5	4	13	0	0	0
NW_001837900.1	7	5	4	8	205	0
NW_001837902.1	4075	3674	4277	1651	510	2433
NW_001837903.1	80	57	92	80	3951	69
NW_001837904.1	2	19	17	4	334	1
NW_001837905.1	29	12	40	0	21	0
NW_001837906.1	7	6	10	3	451	12
